# Supplementary material for: Arbovirus risk perception as a predictor of mosquito-bite preventive behaviors in Ponce, Puerto Rico
Source: PLoS Negl Trop Dis. 2022 Jul 26;16(7):e0010653. doi: 10.1371/journal.pntd.0010653 (PMC9355236; doi:10.1371/journal.pntd.0010653)
Supplement: S1 Table — Model outputs presented in this supporting document provide the statistics that support Figs 1 and 2 in this manuscript. Table A in S1 Table. Model outputs describing the relationship between perceived risk and household expenditure (USD) on mosquito-preventive products, COPA, 2018-2019a. Table B in S1 Table. Model outputs describing the relationship between perceived arboviral risk and the probability of engaging in at least three household-level protective behaviors, COPA, 2018-2019a. (DOCX) [file pntd.0010653.s001.docx]

**Table A. Model outputs describing the relationship between perceived risk and household expenditure (USD) on mosquito-preventive products, COPA, 2018-2019^a^**

|  | **N** | **Multiple Imputation** | **Exposure Coding** | **Low Risk Perception Estimate, β (95% CI)^b^** | **High Risk Perception Estimate, β (95% CI)^c^** | **BIC^d^** | **AIC^d^** |
| --- | --- | --- | --- | --- | --- | --- | --- |
| **Model 1** | 2088 | No | 1 ordinal variable | $15.7 (2.6, 28.7) | $31.3 (18.3, 44.4) | 28210 | 28040.96 |
| **Model 2** | 2088 | No | 2 disjoint indicator variables | $1.6 (-22.1, 25.2) | $28.1 (1.6, 54.6) | 28216 | 28041.00 |
| **Model 3** | 2353 | Yes | 1 ordinal variable | $17.9 (5.8, 30.1) | $35.9 (23.7, 48.1) | N/A | N/A |
| **Model 4** | 2353 | Yes | 2 disjoint indicator variables | $3.8 (-18.3, 25.9) | $33.3 (8.7, 57.9) | N/A | N/A |

^a^ All models included the full set of covariates as described in the methods section.

^b^ Beta estimates in this column represent the average difference in household expenditure (USD) on mosquito preventive products when the household representative perceived their household at low risk compared to when the representative perceived their household at no risk. Positive values indicate increased spending; negative values indicate decreased spending.

^c^ Beta estimates in this column represent the average difference in household expenditure (USD) on mosquito preventive products when the household representative perceived their household at low risk compared to when the representative perceived their household at no risk. Positive values indicate increased spending; negative values indicate decreased spending.

^d^ Multiple Imputation models do not output model fitness statistics. Therefore, the model with the best fit (i.e., lower BIC values) was determined prior to imputation, and the parallel imputed model was then preferred.

**Table B. Model outputs describing the relationship between perceived arboviral risk and the probability of engaging in at least three household-level protective behaviors^a^. COPA, 2018-2019^a^**

|  | **N** | **Multiple Imputation** | **Exposure Coding** | **Low Risk Perception Estimate, β (95% CI)^b^** | **High Risk Perception Estimate, β (95% CI)^c^** | **BIC^d^** | **AIC^d^** |
| --- | --- | --- | --- | --- | --- | --- | --- |
| **Model 5** | 2093 | No | 1 ordinal variable | 0.05 (0.02, 0.08) | 0.11 (0.08, 0.14) | 2831.1 | 2661.7 |
| **Model 6** | 2093 | No | 2 disjoint indicator variables | 0.06 (0.01, 0.11) | 0.11 (0.05, 0.17) | 2838.6 | 2663.5 |
| **Model 7** | 2353 | Yes | 1 ordinal variable | 0.05 (0.02, 0.08) | 0.10 (0.07, 0.13) | N/A | N/A |
| **Model 8** | 2353 | Yes | 2 disjoint indicator variables | 0.05 (0.01, 0.10) | 0.10 (0.04, 0.16) | N/A | N/A |

^a^ All models included the full set of covariates as described in the methods section.

^b^ Beta estimates in this column represent the average difference in probability that the household engaged in 3 or more protective behaviors when the household representative perceived their household at low risk compared to when the representative perceived their household at no risk. Positive values indicate increased probability of engaging in household-level protective behaviors; negative values indicate decreased probability of engaging in household-level protective behaviors.

^c^ Beta estimates in this column represent the average difference in probability that the household engaged in 3 or more protective behaviors when the household representative perceived their household at high risk compared to when the representative perceived their household at no risk. Positive values indicate increased probability of engaging in household-level protective behaviors; negative values indicate decreased probability of engaging in household-level protective behaviors.

^d^ Multiple Imputation models do not output model fitness statistics. Therefore, the model with the best fit (i.e., lower BIC values) was determined prior to imputation, and the parallel imputed model was then preferred.
